# Managing linguistic obstacles in multidisciplinary, multinational, and multilingual research projects

**DOI:** 10.1371/journal.pone.0311967

**Published:** 2024-12-05

**Authors:** Alison Specht, Shelley Stall, Jeaneth Machicao, Thibault Catry, Marc Chaumont, Romain David, Rodolphe Devillers, Rorie Edmunds, Robin Jarry, Laurence Mabile, Nobuko Miyairi, Margaret O’Brien, Pedro Pizzigatti Correa, Solange Santos, Gérard Subsol, Lesley Wyborn

**Affiliations:** 1 Terrestrial Ecosystem Research Network, The University of Queensland, Brisbane, Australia; 2 American Geophysical Union (AGU), Washington, DC, United States of America; 3 University of São Paulo, São Paulo, Brazil; 4 Espace-Dev (IRD, University of Montpellier, University of Guyana, University of La Réunion, University of Antilles, University of New Caledonia), Montpellier, France; 5 Laboratory of Computer Science, Robotics and Microelectronics of Montpellier (LIRMM, National Centre for Scientific Research (CNRS), University of Montpellier), Montpellier, France; 6 University of Nîmes, Nîmes, France; 7 European Research Infrastructure on Highly Pathogenic Agents (ERINHA), Brussels, Belgium; 8 DataCite, Hannover, Germany; 9 French National Institute of Health and Medical Research (Inserm), University of Toulouse III ‐ Paul Sabatier, Toulouse, France; 10 National Institute of Information and Communications Technology, Koganei, Japan; 11 University of California, Santa Barbara, Santa Barbara, CA, United States of America; 12 Scientific Electronic Library Online (SCIELO), São Paulo, Brazil; 13 National Computing Infrastructure, Australian National University, Canberra, Australia; Shanghai International Studies University - Songjiang Campus, CHINA

## Abstract

Environmental challenges are rarely confined to national, disciplinary, or linguistic domains. Convergent solutions require international collaboration and equitable access to new technologies and practices. The ability of international, multidisciplinary and multilingual research teams to work effectively can be challenging. A major impediment to innovation in diverse teams often stems from different understandings of the terminology used. These can vary greatly according to the cultural and disciplinary backgrounds of the team members. In this paper we take an empirical approach to examine sources of terminological confusion and their effect in a technically innovative, multidisciplinary, multinational, and multilingual research project, adhering to Open Science principles. We use guided reflection of participant experience in two contrasting teams—one applying Deep Learning (Artificial Intelligence) techniques, the other developing guidance for Open Science practices—to identify and classify the terminological obstacles encountered and reflect on their impact. Several types of terminological incongruities were identified, including fuzziness in language, disciplinary differences and multiple terms for a single meaning. A novel or technical term did not always exist in all domains, or if known, was not fully understood or adopted. Practical matters of international data collection and comparison included an unanticipated need to incorporate different types of data labels from country to country, authority to authority. Sometimes these incongruities could be solved quickly, sometimes they stopped the workflow. Active collaboration and mutual trust across the team enhanced workflows, as incompatibilities were resolved more speedily than otherwise. Based on the research experience described in this paper, we make six recommendations accompanied by suggestions for their implementation to improve the success of similar multinational, multilingual and multidisciplinary projects. These recommendations are conceptual drawing on a singular experience and remain to be sources for discussion and testing by others embarking on their research journey.

## 1 Introduction

Environmental challenges such as biodiversity decline, climate change, and viral pandemics rarely stop at national, disciplinary, or linguistic borders. Instead they usually demand international collaboration to find convergent solutions [1, 2]. To discover such solutions it is necessary to bring together experts with different, but pertinent, disciplines and skill sets, and to enable access to and sharing of data and information across geographic and cultural boundaries. These requirements were advocated in the UNESCO Recommendation on Open Science in 2021 [3], which also emphasised the need to ensure that multilingual scientific knowledge is openly available, accessible, and reusable. International collaborative research benefits the researcher as well as being for the common good [4], but the lack of a common frame of reference can greatly impede collaboration and often requires the development of new frameworks based on a common language or ontology [5, 6].

Groups of experts are commonly brought together (or come together of their own volition) to pool their expertise and knowledge to generate innovative, novel insights and potentially to achieve solutions [7]. Such diverse, often multidisciplinary, research teams are usually created in response to a problem: members are deliberately chosen with relevant skills and expertise to contribute to a solution, while keeping in mind other factors, such as organisation, country, and life situation [8]. Teamwork of any sort, however, is not simple; effective teamwork does not magically happen and can be impeded by lack of communication among parties for several reasons. These reasons, if identified, can be negotiated and their effects reduced. If not, they can be a breakpoint for the group. The physical separation of group members creates logistic problems, and if collaboration is remote only, time zone challenges can be prohibitive [9, 10].

The information generated to analyse and derive solutions is still siloed in different languages and locations throughout the world [11, 12]. Although English is the most commonly used international scientific language, and having such a language certainly facilitates communication [13], much information of value in biodiversity conservation, for example, is published in other languages and consequently overlooked [13–16]. Taking into consideration the variety of terms and concepts across languages and cultures has been argued to lead to better conservation decisions [16, 17]. Equally, not conveying results in a multiplicity of languages can impede efforts to conserve and protect a species. A lack of recognition of the semantic diversity involved within and across languages undoubtedly affects practice [12–18]. As a means to mitigate such knowledge gaps, it has been suggested that researchers and the scholarly literature should aim to be linguistically inclusive by, for example, providing non-English language abstracts [19, 20] and improving translation tools [11]. Good translations, of course, occur at the concept level, not as a simple word-for-word translation, a limitation of most on-line translation tools [21]. Fundamentally, language is not a precise tool that can be used to accurately express all concepts, but rather a collection of words and phrases that can be used to refer to a variety of different concepts, which can lead to confusion and misinterpretation [22]. Words and phrases have potentially different meanings in the minds of the communicator and the receiver, and their interpretation will depend on the context in which they are expressed. Paying attention to, and asking questions about, the words people use can point to repeated patterns, or touchstones, as well as sticking points that shape collaborations [23].

As multidisciplinarity is becoming the norm in research projects, its emergence is conditioned by the willingness to overcome disciplinary and linguistic barriers. A desire to learn from other fields of expertise is key to shared (convergent) data-driven ways of working, and transformative practices [24, 25]. Communication among team members, and understanding the different practices of each member, is modified by the prism through which each views the world. This can be affected by nationality, disciplinary background or sphere of expertise within that discipline. For effective multinational and multidisciplinary collaboration to occur, each party needs to have some cognisance and understanding of the different ’languages’ used by their fellow researchers, and they often need to engage in active co-interpretation.

## 2 Potential terminological challenges

Researchers from different domains often have their own discipline-specific terminology, consider different types of data more important than others, or deem different kinds of analyses as being more valid [26]. Such barriers can be syntactic (different formats of information), semantic (different groups assign different meanings to information), or pragmatic (different groups have different practices or interests) [27]. Disciplinarily defined language often resides exclusively within a community and is disconnected from upper-level knowledge. If semantic inconsistencies exist but are poorly identified or unknown, they can lead to misunderstanding and misuse, and ultimately impair data management itself [10]. High levels of disciplinary diversity can reduce the functionality of a research group [8, 28], especially if there is significant cognitive distance between the disciplines and insufficient time or willingness among group members to bridge the gaps [24, 29].

As Earth observation data and products increase in availability, it is common for researchers from the computer sciences (data, model sciences or Artificial Intelligence ‐ AI) to use these products in their work. Without appropriate expertise, however, the input data and the interpretation of any analysis can be challenging. The opposite pattern can also occur as more and more computer models are made available and used by people lacking expertise in AI. Modelling in multidisciplinary space requires input from all participants to ensure model validity, relevance, transparency and acceptability [30]. The rewards of overcoming disciplinary boundaries are well recognised with enhanced intellectual stimulation and novel outcomes occurring when interdisciplinary teams function well [31–33].

It has been found that increasing the number of countries represented in a research group results in the degree of innovation in the group being reduced [8]. Participants from different countries with different languages and ways of working can have communication and cultural challenges that take time to overcome [8, 34]. An often overlooked component resulting in difficulties in international collaborations is the different terminology used for in-country administrative units such as provinces versus states, and villages versus towns. Cross-country comparisons depend on the quality of aggregation which can affect the granularity and accuracy of information [35].

Globalisation of research requires interoperability of observations and experimentation systems [22, 36]. Programming and data languages present a challenge, especially for machine discoverability. Developers are often experts in only one programming language (e.g. Python, C++, or R) and it can be challenging to use code that has been developed by another researcher, even in a known language. It is even more difficult to use code developed in an unfamiliar programming language. Increasingly researchers are using (with attribution) code that is openly available and converting it to a more familiar programming language for the purpose of reproducing experiments [37]. The diversity of programming languages, programming styles, and frameworks, even in a familiar computer language, can be very limiting and is certainly opaque to non-experts.

To improve sharing and interoperability of data, software and workflows across research teams, common FAIR (Findable, Accessible, Interoperable and Reusable) vocabularies that are both human and machine-readable have been proposed [38, 39]. These are intended to enable data interoperability and meta-analyses, even when data or software have different origins and are based on multiple vocabularies (as per FAIR Principle I2) [38].

## 3 Rationale

**Fig 1:**
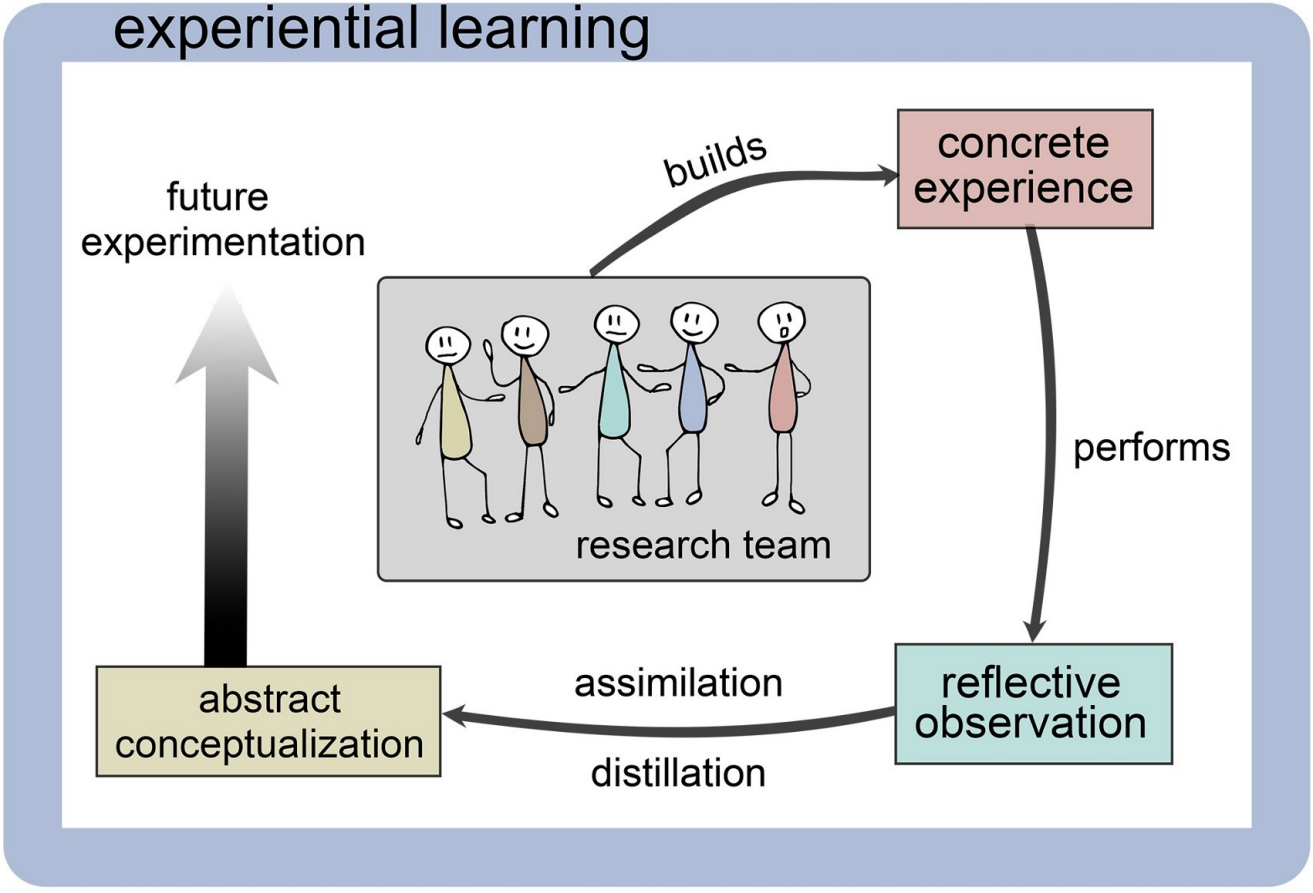
Model of the experiential learning process.

Adapted from [44, 45]

Learning from experience (experiential learning) is a fundamental way teams improve their practice. Observation and reflection on the root causes of problems and dysfunctional workflows can result in adjustment of practice, and new implications for action can be drawn [40, 41]. Individuals in software development teams learn during the work by reflecting on the process (reflection in action), but also after the work is either fully or partially completed (reflection on action) [42]. Continual adjustment of practice in response to internal and external feedback is fundamental to Agile Project Management, a well-accepted method of experience-based modification of practices in the software community [43]. Too often, however, work practices are adjusted ‘on the run’ and little is learnt from the experience for future reference [42]. The experiential learning process for a research team is illustrated in Fig 1. This is in contrast to the pattern for industry, for example, where the process becomes a repeated cycle, in each case the next iteration benefitting from the learning of its predecessor. Research projects usually finish when the funding runs out, so it remains for others to take advantage of the learning gained.

In this paper we use participant observation and reflection of their research experience as our research strategy [24, 40, 46]. We use a case study strategy, ideally suited to in-depth exploration of phenomena and for constructions of theory in its real-life (natural) context [4, 46, 47]. Our exploratory case study (sensu [48]) consists of two workflows within a multi-disciplinary and multinational research project. The research questions we posed are: (a) what are the terminological obstacles encountered, (b) are mismatches in language concepts due to disciplinary differences, native language, or country-specific nomenclature, and (c) what is the impact of these obstacles on the two workflows. From this structured experiential learning we propose ways to anticipate and overcome similar terminological stumbling blocks for future testing.

## 4 Case study

We examine the experience of two teams within a technically innovative, multidisciplinary, multinational, and multilingual research project aiming to be an exemplar of Open Science practices, the PARSEC project, ‘Building new tools for data sharing and reuse through a transnational investigation of the socioeconomic impacts of protected areas’ [49, 50]. PARSEC was an international Belmont Forum-awarded research project under a Coordinated Research Action on Science-driven e-Infrastructure Innovation. The 38 team members and six associate members were drawn from Brazil, France, Japan, the United States of America, and Australia. Two postdoctoral researchers and several short-term researchers were funded through the project. PARSEC ran from 2019 until 2024—notably encompassing the Covid19 travel restrictions—and was divided into two interrelated components: one pursuing a scientific objective, the other pursuing e-infrastructure innovation according to Open Science principles.

The core scientific investigation (carried out by 25 ’**Synthesis Science Strand’** members) examined the socioeconomic effects of creating marine and terrestrial protected areas. This approach required combining and analysing existing remote sensing data with existing socioeconomic data using AI and other tools. The goal was to develop robust methodologies capable of estimating trends in socioeconomic indicators from remote sensing imagery. The 13 **’Data Science Strand’** members were charged with developing recommendations for data and code curation through interaction with the Synthesis Science Strand team and the wider research community. The goal was to remove barriers to data reuse by promoting best practices throughout the research data life cycle compliant with Open Science principles.

The disciplinary range in PARSEC was considerable, with specialists in data science, research data management, remote sensing data analysis, AI, machine learning, Deep Learning (DL), spatial systems, socioeconomics, wildlife biology, and ecology. The majority of team members had experience working with a variety of data types, from observational, spatial, remotely sensed to socioeconomic data. Most team members had experience working on more than one type of data. Half of the team members at the beginning of the project had limited experience in Open Science data practices [51].

The work of the Synthesis Science Strand started with an assessment of the use of Convolutional Neural Networks (CNN) to estimate poverty in a sample of east African villages [52], and an exploration of using Google Street View to detect socioeconomic conditions in an area of Brazil using DL methodology [53]. Code developed for the project has been made openly available [54, 55]. The Data Science Strand started work with a general introduction to the FAIR Principles and the steps required to account for them throughout a project [56] and examining best practices for reproducibility of DL experiments [37]. The Data Science Strand established various tracksheets for data and software use and for outputs and a project ’dictionary’ that was shared in a common on-line workspace.

Challenges due to the international and multidisciplinary nature of the work occurred (a) in the development and application of the DL model, and (b) in the preparation of guidelines for our multilingual researchers. The experience of the teams facing these two challenges are the focus of this paper and they will be referred to as the ’DL’ and ’Checklist’ teams.

### 4.1 DL team

The DL team was dedicated to leveraging advanced machine learning techniques, particularly DL models, for the analysis of remote sensing imagery in Brazil, Japan, and several countries in east Africa. The six DL team members were situated across France, Brazil, and North Africa. None of the DL team members were native English speakers, but all were computer scientists or engineers with high-level expertise in the use of AI to elucidate patterns across different types of data. The team used the English language to collaborate. Five of the members are authors of this paper (MC, PPC, RJ, JM, GS).

**Fig 2:**
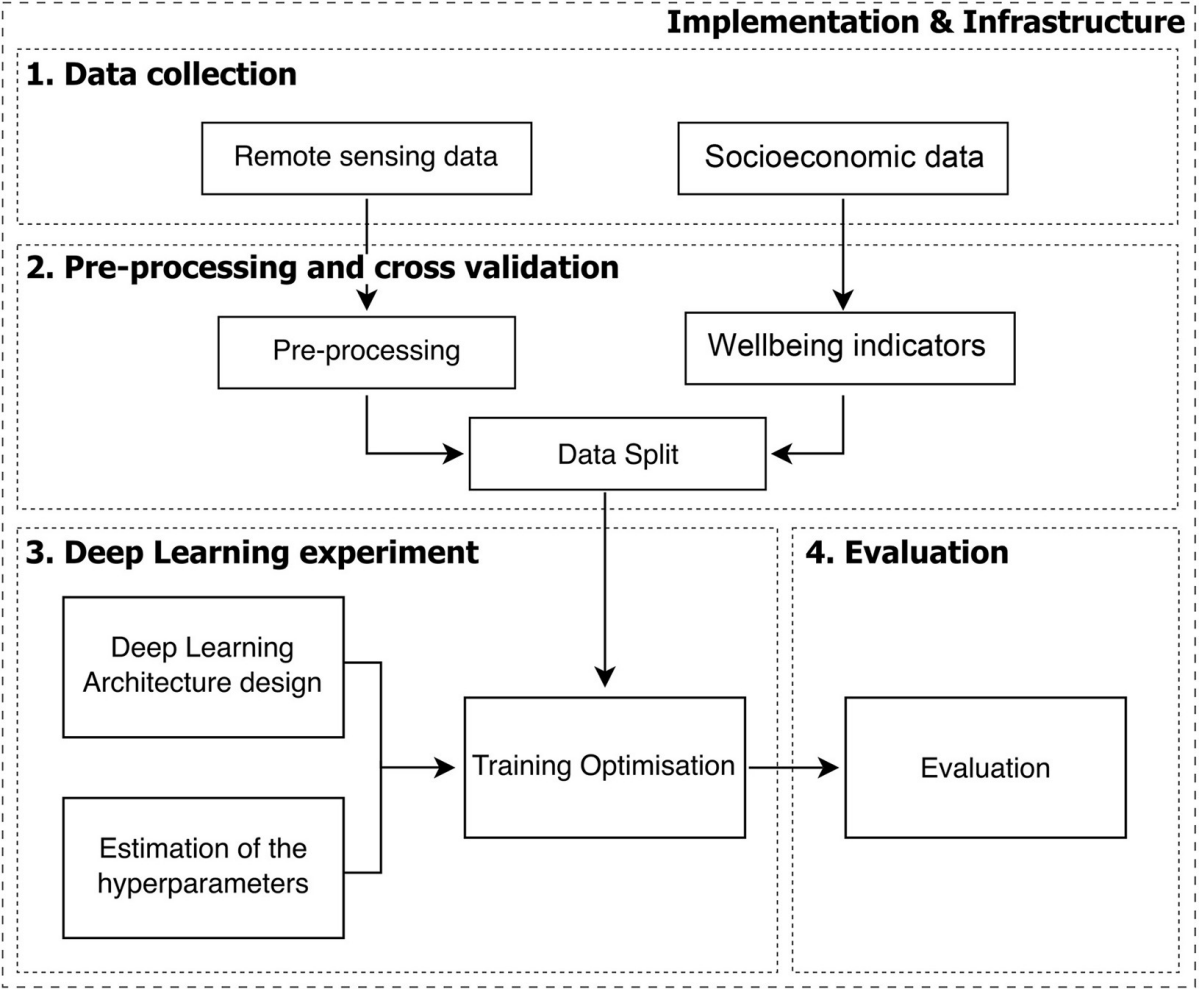
Flowchart for socioeconomic wellbeing estimation using remote sensing data and DL approaches. The general components are in bold while the steps of the DL workflow are shown in the boxes (adapted from [37]).

At the start of the project there were various meetings and workshops across the whole PARSEC team to understand the main goals. The most important task was to collect data so that a first data exploration could be conducted. Given the complexity of DL techniques, a generic workflow was adopted by the DL team (Fig 2). The stages of the workflow were:

1. Data collection in which remote sensing and socioeconomic data are gathered for training and validation;
2. Pre-processing and cross-validation tasks, such as data cleaning, integrating poverty indicators, and splitting the data for validation;
3. DL model experiments tailored to our specific objectives; and
4. Evaluation to assess the performance of the DL models, ensuring robustness and efficacy in analysing remote sensing data for socioeconomic estimations.

### 4.2 Checklist team

The Checklist team was composed of a PARSEC Data Strand team member from each funding country (Brazil, France, Japan, and the United States: SSantos, LM, NM and SStall respectively). This team comprised members with considerable expertise in journal publication (i.e. SCIELO, AGU), methods of attribution and provenance (e.g. ORCID), all underpinned by data management expertise. As the PARSEC common language was English, the Checklist team used English as its primary language.

At the time the Checklist team was forming, and the format of the Open Science guidance was being considered, the team converged on the concept of a concise list or set of tasks—a Checklist—that could be quickly understood by a researcher. The team had collectively found that this technique was effective in providing guidance and helping researchers learn and implement new practices efficiently. Checklists are recognised as useful support tools in the implementation of complex processes, helping to summarise information in a concise manner by breaking them down into a series of clear and actionable steps [57]. In PARSEC they needed to be as generic as possible for the scientific and technical elements to be relevant to the wider scientific community.

**Fig 3:**
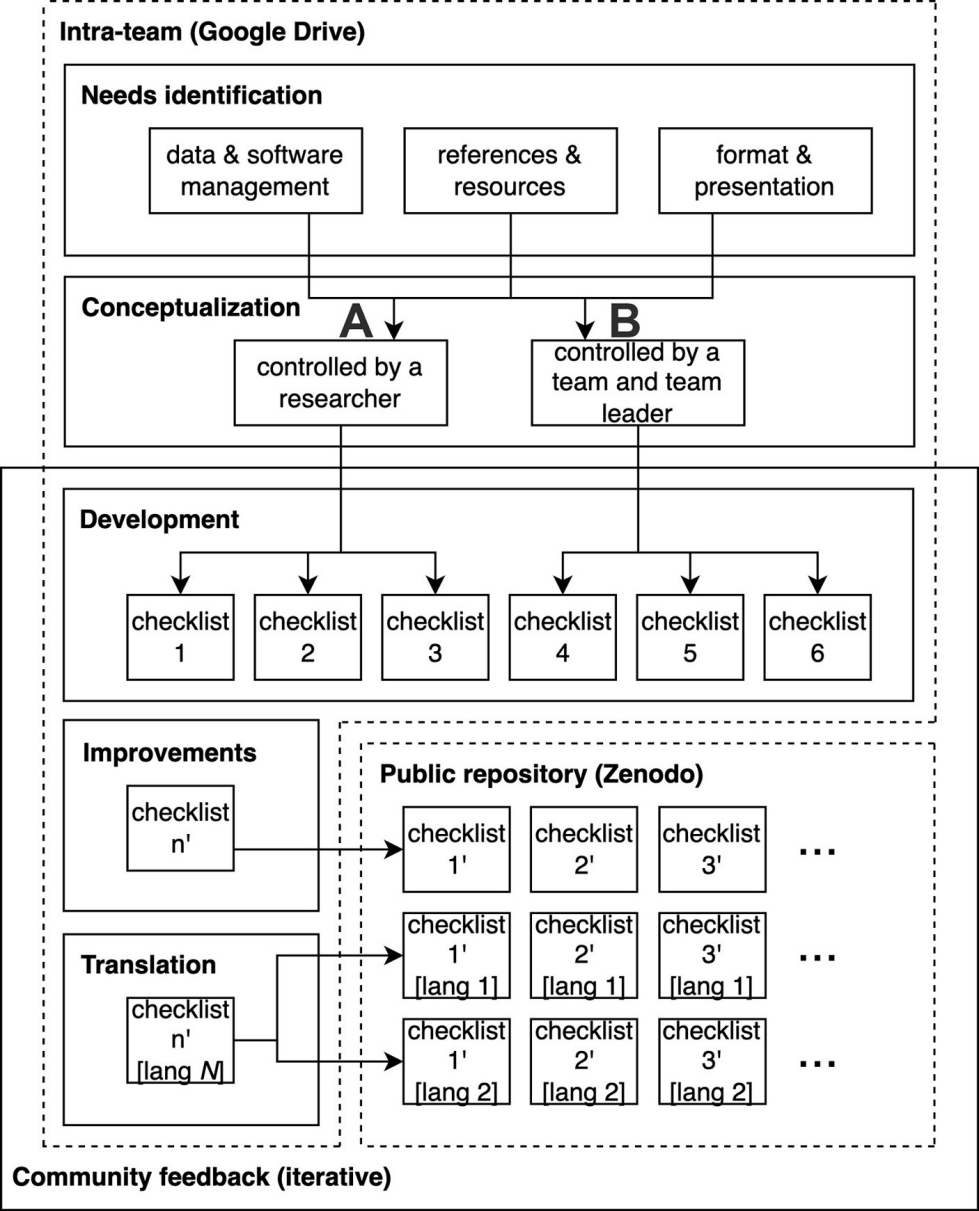
The PARSEC checklist development workflow. This workflow shows the main steps to develop and translate each of the checklists for both level A and level B: determine team needs; iterate on the conceptualization; develop each checklist; translate each checklist; publish, promote, and receive feedback.

Working iteratively, and informed by experiences with the PARSEC Synthesis Science Strand, the team organised the elements of Open Science specific to data and software management into two levels and produced three checklists for each (Fig 3):

- Elements that a researcher can control directly (1: data presence, 2: data documentation, and 3: software documentation)
- Elements needed by a research team and their leader (4: open science practices, 5: resources and guidance, and 6: digital objects preservation).

The expertise of one of the post-doctoral team members, a native Spanish speaker, was harnessed to provide initial input. Updates were made in consequence to this and feedback from other PARSEC members. The initial English version of each checklist was published in the PARSEC Zenodo community [58–64]. The completed checklists were promoted through the Data Science Strand’s networks and the feedback received informed revisions of each checklist which were then re-published in Zenodo under version control (Fig 3).

After strong positive feedback was received about the English version of the checklists, it was clear that translations into other languages would be optimal (consistent with [12]). As the Checklist team had native speakers of French, Japanese, Portuguese and Spanish, these languages were our starting point. Each team member leading the translation consulted with someone local to them who could validate it. Questions about context were brought back to the project team and clarifications made, including tracking updates to the English version (Fig 3).

Each checklist was reviewed by the larger PARSEC team and feedback was incorporated. As the translations were completed, they were published on Zenodo [58], and promoted on social media and at relevant conferences. The team collected any additional feedback for further updates.

**Fig 4:**
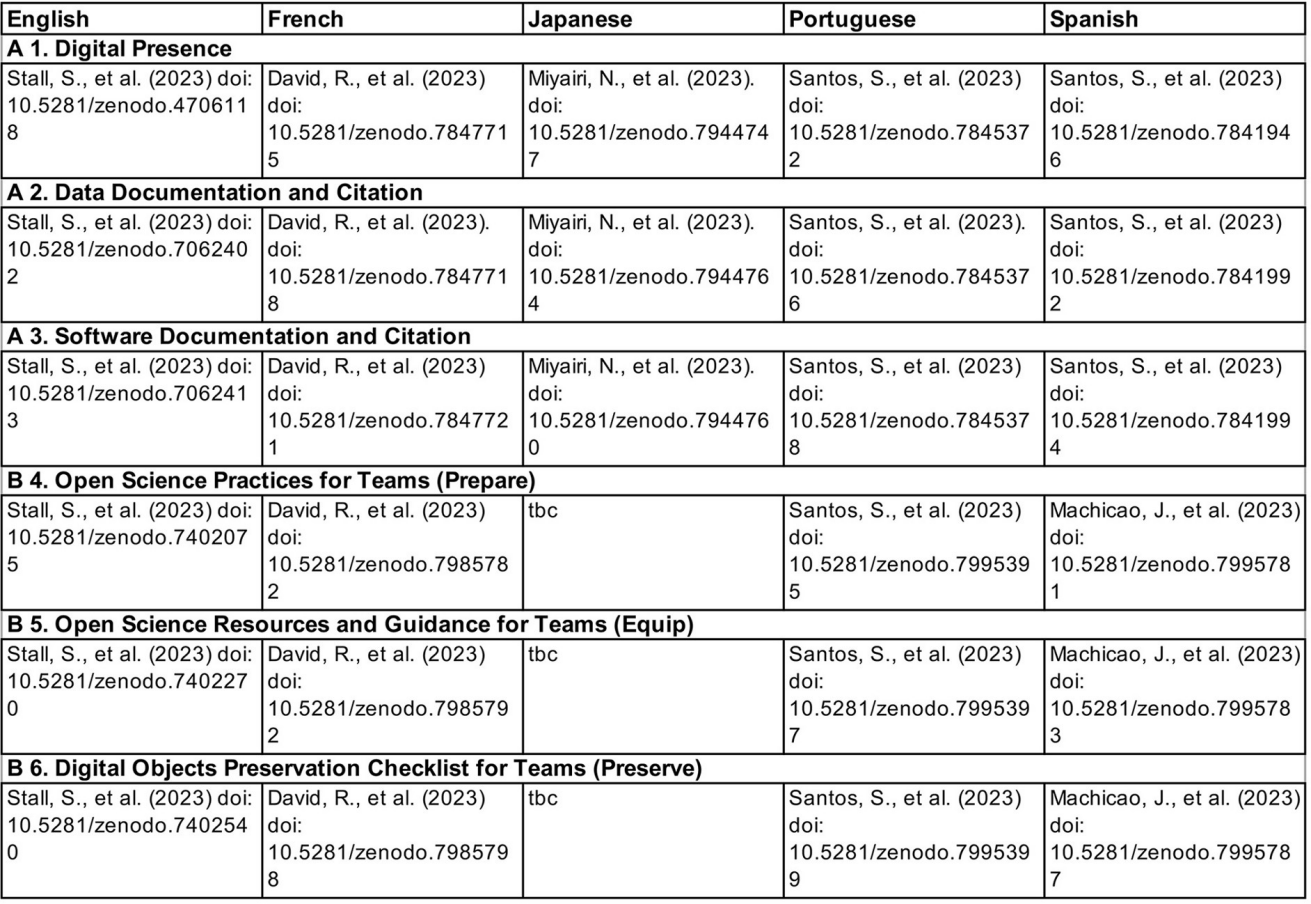
Checklists produced by the PARSEC checklist team for researchers and research teams. Full citations for the English versions are in the references. TBC is an abbreviation for ‘to be created’.

The French [64–70], Japanese [71–73], Portuguese [74–79] and Spanish [80–85] checklists at the time of writing this paper are shown in Fig 4.

## 5 Methods

Each team was asked to reflect on its practices, (a) identifying terminological obstacles it encountered in its workflows, (b) to classify them in one of the nine ways shown in Table 1 (inspired by David et al. [21]), and (c) to categorise them on a five-point scale of importance, from 5, indicating a challenge that stopped progress, to 1, a challenge that was easily overcome. The teams did not include a professional linguist but the terminology made sense to us.

**Table 1:** Potential terminological challenges.

code	Categories of linguistic confusion
A	Polysemic (1 term or concept, multiple meanings), a form of ambiguity [86 p.89]
B	Linguistic confusion due to (apparent) multiple terms for a meaning (i.e. synonyms)
C	Fuzziness in language or terminology matches (existing and evolving nuances), a form of ambiguity [87]
D	Idiomatic language terms adopted from another language with different contexts, a form of ambiguity [86 p.7]
E	Disciplinary domains (communities of practice) assign different meanings to the same term [88, 89]
F	Country of practice or origin may assign different meanings to the same term [90]
G	Vagueness: involves uncertainty about the actual meanings of particular terms, e.g. administrative units differ in name and nature from region to region and country to country [86 p.83, 91]
H	Lexical gap: New concepts (words, phrases) that don’t exist in the target language [92]
I	Adoption challenges: a ‘new’ term needs to have community endorsement for it to be recognised and used and this has not happened yet [93, 94]

The DL team reflected on its practice, guided by its workflow (Fig 2). Feedback was achieved by completion of a table, supported by a series of virtual meetings. The results were analysed by team members, feedback was obtained, and the table updated. The Checklist team examined the steps in its workflow (Fig 3) through a series of meetings, identifying and describing the stumbling blocks encountered.

## 6 Reflective observations

The two teams presented their observations in different ways, reflective of their different task-sets.

### 6.1 DL team

From initial discussions it became clear that misunderstandings and difficulties in the use of terminology specific to DL methodologies and processes were important. Team members felt strongly that their work was hampered by poor inter-domain understanding of the aims of the project, the scientific question, the hypothesis, and the DL methodology. This led to confusion and lack of useful data provision, causing difficulties in DL model training, estimation, and validation. Team members from different countries had different understandings and norms.

The data collection phase (Fig 2) required cross-team collaboration, both for the acquisition of satellite imagery and socioeconomic data and to optimally align the two. The two relevant disciplinary groups (spatial and socioeconomic) had to work with the DL team to select appropriate data for the analysis, including the time range and the frequency of repeat observations, while ensuring the specificity of each country’s organisational structures was accommodated. This required more effort and time than was initially expected. Often available data were in a non-English language with no translation available, which prevented their use and stopped the investigation completely. As Demographic and Health Survey data were not available in all the study countries, a robust and comparable poverty (or wealth) index had to be created, requiring advice and participation from across the team. Once these matters were in place, the actual DL modelling phase was straightforward. The evaluation stage, as the last step of the workflow, again required an integration of ideas and communication with the full team to understand the meaning of obtained results (the spatio-temporal mapping estimations).

When reviewing the occurrence of terminological impediments, four workflow stages were noted: (i) definition of the work, (ii) collection of both remotely-sensed imagery and socioeconomic data, (iii) pre-processing of imagery and (iv) data splitting for cross validation. These workflow stages highlighted the lack of explicit methodological description in many publications (Table 2). Overall, eight obstacles were identified, with most clustered around challenges concerning the whole team’s understanding of the DL work, the acquisition of population data for the project, and incomplete or inaccurate terminology and metadata in resources (e.g. exemplar articles and data sources).

**Table 2:** Terminological challenges found by the DL team as it executed its work.

Stage in workflow	Obstacles encountered * high (5: stops the work) to low (1: minor interruption)	Examples and typology ^#^
**1.** Definition of the work (scientific question, hypothesis, methodology) across the team	a. Alignment of principal keywords from ecology across domains [5]	The term ’**ecoregion**’ was misunderstood. [E] Disciplinary domains
	b. Understanding (alignment) of the DL model by all the team was lacking [2]	Confusion between the meaning of the terms **’prediction’** and **’estimation’.** [B] Linguistic confusion
	c. Regional/national key development questions were differently understood by team members [2]	The terms **’survey’** and **’census’** were used differently across the project (and in the literature) and the difference between these terms was not initially appreciated. [B] Linguistic confusion, [E] Disciplinary domains, [F] Country of practice
	d. Setup of the terminology for the methodology presented challenges [3]	Use of various terms, e.g. ’**control ‐ reference’**; ’**treated ‐ non-treated**’. [C] fuzziness in terminology ’**mirror ‐ control**’ [D]- Idiomatic terms **’experiment ‐ run’** [A] Polysemic; [E] Disciplinary domains
**2.** Data collection: census or survey	a. Difference in data organisation between different organisations or institutions [3]	**’prefecture ‐ state ‐ province’; ’municipality ‐ city’; ’census tract ‐ census sector ‐ block’; ’village ‐ town’; ’enumeration area ‐ ground truth sample area’** [B] Linguistic confusion, [G] Vagueness
	b. Opaque and unfamiliar socioeconomic terminology [3]	**‘survey ‐ census’; ’panel ‐ population sample’; ’wave ‐ round ‐ repeat instance’** [E] Disciplinary domains, [C] Fuzziness in terminology
**3.** Pre-processing of imagery	a. Confusion with some terminology [1]	Use of various terms for the same thing **’nightlight ‐ night-time lights’; ’daytime imagery ‐ multispectral ‐ diurnal imagery’**. [B] Linguistic confusion
**4.** Data split for cross-validation	a. Exemplar articles do not always follow correct procedures, or state their methods clearly or fully [3, 4]	**’test (regular) ‐ out-of-domain test’, ’in-country’, ’out-of-country’** [C] Fuzziness in terminology, [I] Adoption challenges

* Each obstacle was ranked using a 5-point scale according to its seriousness, from 5 for high (stopped the work proceeding until a solution could be found) to 1 for a minor interruption. The nature of each obstacle was classified following the 9 criteria.

# The key to the linguistic categories [A] ‐ [I] is found in Table 1.

Some of the linguistic (or terminological) confusion arose during the conceptualisation and establishment of the experiments as a result of disciplinary differences, such as the use of the word ’ecoregion’ (1a, Table 2). This term was assumed by the DL team to refer to an abstract model for the work, the DL team being unaware of its well-accepted use in environmental science, and its basis for the Synthesis Science Strand’s work [95, 96]. Once this was explained, the methodology for selecting sites had to be revised. Similarly, the term ’experiment’ proved to be quite a stumbling block as it had a different meaning for computer scientists than experimental scientists (1d, Table 2). In both cases discrepancies were not realised until after some of the work had been completed and were exposed when sharing with interdisciplinary team members from other parts of the PARSEC project.

At a more practical level, the enumeration area was often not only named differently from country to country but also differently defined spatially (2a, Table 2). For example, population size as a social clustering tool was used for collection and delivery of data in many countries, which could mean different cluster types from one location to another. This created some difficulties for the remote sensing experts on the team when comparing physically different and administratively (geopolitically) different areas. In addition, names and designated areas were sometimes dynamic over the study period.

Several terms necessary for incorporating data into the DL model were different from country to country (2b, Table 2); for example, the methods and terminology used to obtain socioeconomic data. In some methods, data from a sample of people (a panel) were used and re-surveyed over repeated instances (waves), and in others, data were obtained from whole country surveys (a census). The frequency and duration of data collection was also different across countries (e.g. every 5 years versus 10 years). An additional issue was that, in several cases, terms were used somewhat casually by team members, and differences between such terms were outside the DL team’s knowledge. Realising this required active and constant dialogue among team members, which in a distributed team across multiple time zones was not always easy to achieve. The use of varying terminology for the same concept (linguistic confusion [I]), not just within the team but more broadly in the referenced literature, was an obstacle that also had to be overcome (3a, Table 2).

Two additional confusions arose when explaining the constraints of the DL method to non-DL specialists in the team (4a, Table 2). In training a DL model, the test sets and the training sets must not overlap, else one will get a positive bias. For instance, X is a dataset split into X_train and X_test. The model is trained on X_train and evaluated on X_test, and a performance score is computed. This is referred to as a ’test (or regular test)’. Then, it is often argued that the model can be used on another dataset Y. It can be, but sometimes the underlying message is that the performance score on the dataset Y and X_test will be similar. To prove the last statement one could use an ’out of domain test’. In a related confusion, an exemplar paper used the terms ’within/in-country’ and ’out-of-country’ without explanation.

Impediments to progress were particularly acute at the beginning of the project and they returned somewhat at the end. An assumption was made that ’everyone knows this’ and it was clearly not the case for the DL team. Similarly, casualness and variability in terminological use in important and relevant literature produced significant delays; some could be solved by speaking with the authors, but that was not always possible (4a, Table 1). Basic across-team misunderstanding generally produced challenges (1d, 2a and 2b, Table 1). Developing a common cross-country suite of terminology and definitions for the DL team to enable its model to be applied was time consuming (2a, Table 1).

### 6.2 Checklist team

The goal of the Checklist team was to ensure that researchers reading in their native language would easily understand the concepts and recommendations included in each checklist. In short, to make Open Science easy to adopt in daily practice. Because the concepts and practice around Open Science are still new, identification of language-specific references was difficult. Many sources were used to support the translations with the expectation that on-line translation tools would be helpful, but they were not completely so.

#### Concepts without existing equivalent words

The English language has been historically influenced by Latin languages, borrowing many of its words (e.g. ’Data’ is Latin, ’Management’ comes from old French, as does ‘Plan’). This linguistic proximity and the format of the scripts used facilitate translation between English and the Latin-based languages of French, Portuguese and Spanish. For languages based on other roots or where the basic form is an ideogram, the translations from English are less direct. For example, the phonetic (katakana) transcription for the term ‘Data Management Plan’ (データマネジメントプラン, deetamanijimentopuran) was used, as there was no Japanese equivalent term with this meaning. Specifically, the word is directly ‘borrowed’ from the English. If the equivalent kanji was used (which in theory it could be), it would be a lengthy and confusing term. In contrast, the concept of ’Data Management Plan’ can be directly mapped in French to ’Plan de gestion des données’. Another example encountered was the English term ’funder’. The verb suffix ‘-er’ indicates someone or an organisation that ‘does something’, but in Japanese ‘funder’ is expanded as 研究資金配分機関, meaning ‘organisation allocating research funding’. It must be explained literally.

Nature of issue: [H] lexical gap; [C] fuzziness in language. A minor interruption [1].

#### Concepts that exist but do not have a routine translation

In French the strict translation of the word ‘checklist’ would be ’liste de vérification / contrôle’ which is not usually used in practice. As the English term ‘checklist’ is known in the French language, and to be consistent with the easy-to-use and evocative character of the checklist terms, we determined that we should choose a distinctive name; namely, ‘check-liste’.

Similarly, in Japanese, words derived from foreign languages usually make sense if they are written in katakana with a close pronunciation. For example, the phonetic translation for ‘computer’ is コンピュータ (konpyuuta) and ‘notebook’ is ノートブック (nootobukku). However, when new terms are generated by simply combining existing words, such as ‘computation(al) notebook’ to describe something specific (in this case applications such as Jupyter Notebook and R Markdown), doing the same in Japanese would not work. For this reason, the translation of ‘computation(al) notebook’ into Japanese had to use the redundant phrase 計算機上のノートブック環境, a ‘notebook environment on computers’.

Nature of issue: [C] fuzziness in language; [F] country of practice; [H] lexical gap; [I] adoption challenges. A significant interruption [3] and clarifications had to be made.

#### Translations exist, but they need social endorsement for adoption

When practising Open Science, being inclusive includes taking the time to ensure that a new concept is fully understood and used by others in their language. This applied to both the checklists being developed, as well as the concepts being used. If a language did not have the words broadly accepted in a relevant community to describe a concept, care had to be taken in the translation process. If the concept needed a concise word or phrase that was not yet widely recognised, the word would be introduced in the language along with a description.

An example is the expression, ‘Digital Presence’, which is emerging alongside increasing online availability of research products. Researchers need to adjust their practices to take advantage of this opportunity, and thus create a ‘Digital Presence’. In French a ‘Digital Presence’ can be translated as ‘Présence numérique’ which is broadly understood but not yet a common expression in all disciplines of science. Until a research community endorses the new term it will not be widely accepted or adopted.

The Japanese translation of ‘Digital Presence’ fell into this same category (vaguely known but not widely adopted), and we decided to use the katakana デジタルプレゼンス (dejitarupurezensu) to simply mimic the pronunciation as it would be more likely to attract the reader’s attention and be remembered as a concept.

Nature of issue: [H] lexical gap; [I] adoption challenges. A significant interruption [3] and clarifications had to be made.

A compendium of the words and phrases used in the checklists is shown in Table 3. The table illustrates that different methods for translation need to be used to ensure the best comprehension of important words. This may include a decision to create new words or ‘borrow’ words from another language. In Japanese a phonetic transcription is used when words are borrowed. The acronym for Data Management Plan, ‘PDG’, is the same for the Latin-based languages (Portuguese, Spanish and French) while ‘DMP’ is used for English and Japanese. In the latter case this is due to the English words being borrowed as shown by the phonetic transcription.

**Table 3:** Examples of the decisions made for Open Science vocabulary translations.

English	Portuguese	Spanish	French	Japanese
Data Management Plan (DMP)	Plano de Gestão de Dados (PGD)	Plan de Gestión de Datos (PGD)	Plan de Gestion des Données (PGD)	データマネジメントプラン (DMP) [phonetic transcription]
Computational Notebooks (e.g., Jupyter Notebook, R Markdown)	Cadernos computacionais	Cuadernos computacionales	Notebook (in routine) / Carnets de notes pour logiciel / Calepin électronique	計算機上のノートブック環境 [English: Computational Notebook Environment]
Lab notebooks	Cadernos de laboratório	Cuadernos de laboratorio	Cahiers de laboratoire	電子ラボノート [English: Digital lab notebooks]
Digital Presence	Presença Digital	Presencia Digital	Présence numérique	デジタルプレゼンス [phonetic transcription]
Open Science Journey	Jornada pela Ciência Aberta	Viaje por la Ciencia Abierta	Parcours Science Ouverte	オープンサイエンスの旅
Persistent Identifier (PID)	Identificador Persistente (PID)	Identificador Persistente (PID)	Identifiant pérenne (PID)	永続的識別子 (PID)

## 7 Conceptualization

As mentioned in the Introduction, multinational, multilingual, and multidisciplinary collaborations require that each party understands the different ’languages’ used by their fellow researchers. We posed the following research questions: (a) what are the terminological obstacles encountered, (b) are mismatches in language concepts due to disciplinary differences, native language, or country-specific nomenclature, and (c) what is the impact of these obstacles on the two workflows. There were indeed several unfamiliar concepts within and outside each of the teams that needed to be translated or explained with variable success. This affected team communication, the acquisition and interpretation of data and information, communication with the wider community, and ultimately in generating outputs and products. The retrospective linguistic classification of the terminological obstacles (A-I) assisted our insight into the actual nature of the difficulties we faced and provided us with a pathway to solutions. Being aware of these potential confusions at the start of the project might have enabled us to anticipate them and to respond more efficiently when they occurred.

The two sub-teams (’DL’ and ’Checklist’) studied in this paper identified different **terminological obstacles,** albeit with some commonalities. Fuzziness in language, disciplinary differences, linguistic confusion, lexical gaps and adoption challenges were most often cited as the cause of terminological mismatches. Some of these obstacles significantly impeded progress, such as the lack of terminological equivalents across disciplinary domains or languages. Some obstacles were less significant, but required attention if project goals were to be achieved, such as terminology used in one domain that was not familiar to those from another, or adoption challenges when communicating new concepts in a country. Some terms were ’known’, but not yet sufficiently accepted to be translated smoothly. The technical nature of many of the terms and concepts made translation challenging unless the translator had disciplinary insight. Terminology and approaches to nomenclature unique to one country were on occasions surprising and required adjustment, taking time and effort in addition to the main work. Disappointingly, given the expected high standards of scholarly publishing, obstacles occurred due to key terms on which research was based not being defined in the source literature.

Often obstacles took some time to overcome depending on the cognitive, linguistic or developmental distance involved. The cognitive distance across the team was not insignificant; for example DL programmers worked directly with human geographers, a considerable disciplinary difference. There was a joke within the team that for one particular concept, where the word used was the same in two disciplines but had a different meaning in each, that it took ten attempts to explain the difference. It was discussed that the number of times an explanation had to be made could form a scale of difficulty that could be applied to all. As mentioned in the description of the case study, the Data Science Strand team created an on-line ’dictionary’ in the shared workspace at the beginning of the project that was intended as a place where everyone could record terms and definitions, but it was not used. Retrospectively this resource could have alleviated some confusion if team members were (a) more aware of its existence, and (b) contributed to it.

While engaged in the reflective observation process illustrated in this paper, we noted that the use of English as our common working language was essential, but team members had varying levels of English proficiency. This can affect the degree of collaboration and the development of trust [34, 97]. It was observed that although all communications were made in English, it may not have been ‘standard’ English in each case. A ‘common’ version of English was often established that had elements of the mother tongue of each interlocutor. This adjustment was unique to the task at hand, dependent on the team members involved, and not meant to be shared outside PARSEC. This lack of standardisation in language also occurred across disciplinary boundaries, as parties listening to one another interpreted meanings according to their own perspective (Enryo-Sasshi: [98]). The translations by the Checklist team were designed to alleviate this effect for the external research community.

The complexity of translating research concepts into the five main languages in this paper shows how difficult concept-based translations can be. The illustrations provided by Ducarme et al. [17] and Droz et al. [16, 18] for the different meanings conveyed by the concept of ‘Nature’ in a range of languages are evidence of this difficulty. Good translations occur at the concept level, not as a simple word-for-word translation [21], a limitation of most on-line translation tools, and possibly also a limitation of many online domain-specific translation tools. Vanderbilt et al. [6] tested an automated translation of metadata using Google translate, Bing and World Lingo Version, none of which were close to being completely correct. Back translation showed a 60% accuracy for Japanese or Chinese to English, but 90% for Swedish to English. Tools have improved since then, certainly, but caution still needs to be applied.

One of the most important realisations from this work was the need to ensure that all team members had a strong common understanding of the project purpose, good knowledge of the tasks to be done, the requirements for these tasks, and their contribution to them. This should be established at the beginning of the project and revisited throughout the project (the ’redundancy’ of Podestá et al. [99]). In PARSEC full team meetings were held every six months, but more regular conversations were clearly beneficial, especially around particular tasks (as practised within the Data Science Strand). The importance of these meetings was not appreciated by all team members at the time, but not only did it enable the development of trust among team members but also kept conversations alive. It is, as is often the case, only in retrospect that the value of such diligence occurs.

Although co-design of research is commonly a practice mentioned in projects where members of a wider community are involved (i.e. stakeholders who are non-scientists, such as citizen scientists and indigenous knowledge-holders) [3, 100], it is clearly an appropriate model here. In both case studies—the e-informatics research of the DL team, and the communications work of the Checklist team—members from other disciplines or native speakers of other languages, were able to provide checks and clarifications in a timely manner, even if not involved in the core work of a particular team, and thus avoid delays.

## 8 Learning outcomes

The objective of this paper was to highlight, through an empirical approach, the pitfalls of ignoring sources of terminological confusion in technically innovative, multidisciplinary, multinational, and multilingual research projects. Our case study provides unique insight into the effect of terminological obstacles on project work in real time. It confirms much previous understanding but adds a perspective from lived experience. Guided by a classification of terminological obstacles (A-I), our two contrasting research teams reflected on the nature of the terminological obstacles encountered, and the effect on their workflows. This paper not only reports their reflection but also distills their observations into guidelines that could prove useful to others attempting such a journey.

Three different situations emerged from our research experience: (1) concepts without existing equivalent words, (2) concepts that existed but did not have a routine translation, and (3) cases where translations existed but needed social endorsement for adoption. Fuzziness in language, linguistic and disciplinary confusion, lexical gaps and adoption challenges were most often cited as obstacles across both workflow-types. Linguistic distance, when translating new concepts associated with open science across countries and languages, was noted. Practical matters of international data collection and comparison included an unanticipated need to incorporate different types of data labels from country to country, authority to authority.

We thus make the following simple but practical recommendations to improve the success of multinational, multilingual, and multidisciplinary projects (Table 4). These recommendations are conceptual drawing on experience through a reflexive approach (Fig 1) and remain to be sources for discussion and testing by others embarking on their research journey.

**Table 4:** Conceptualization of outcomes.

Recommendation	Practical suggestions for achievement
1. Ensure all team members have a strong common understanding of the project purpose, good knowledge of the tasks to be done, the requirements for these tasks, and their contribution to them. This needs to be revisited as the project evolves.	Spend time at the beginning of the project to introduce the project and post relevant documents (like grant proposals) into a shared on-line space. At regular intervals throughout the project revisit the aims and modify as necessary. Ensure people have a clear idea of their role and tasks in the project, and record these in the shared on-line space to which everyone has access.
2. Start every working group with a dictionary conversation. Define the terms you will be working on and with and record them in a shared space. Continue to update and modify the dictionary throughout a project.	At the beginning of the project spend time ensuring key words and phrases relevant to each main task within it are documented, defined and shared with the whole team (later in the on-line space). Encourage the team to own this information so they feel free to add any new terms as they work through the project. Have a conversation about this at regular team meetings.
3. Never assume understanding across boundaries. The hearer does not always interpret the message in the way the deliverer intends. Take time to check your point is received as you meant it.	Misunderstandings will emerge during project execution. Project leaders can ensure regular ‘show and tell’ of project work within the team, which will maximize the chances of such misunderstandings being identified. Having an active dictionary can assist. Any presentations to the group should be deposited in the shared online space so members can access them for later reference.
4. Think constructively about obstacles encountered, examine them, and share with the wider team. Solutions can be found sooner rather than later.	Within-project conversations need to be regular and, as for recommendation 3, sharing across the team needs to be open. To do this team members need to have developed a trusting relationship, which can be enhanced in regular meetings but also by sharing more relaxed discussions and activities. Obstacles that stop work might include access to data, algorithms that don’t work, and reporting in different languages and jurisdictions. Other team members can provide sometimes unanticipated help!
5. In a complex multilayered project, ensure relevant ’non-core’ participants are involved in the work and have opportunities for meaningful input.	This recommendation pertains to internal and external participants. Internally it is natural for team members to gravitate to their own disciplinary or language groups, but in a complex project with a need for input from a range of expertises and background, team members will be needed to contribute to other areas than their own. A sense of inclusion can be enhanced by the development of trust and regular opportunities to share experiences and progress. Invitations to meetings and workshops need to be clearly sent (via an agreed project mode or a variety of modes) to all team members so they feel welcome and valued. Increasingly research teams do not work in isolation from their communities of practice or indeed the wider community of stakeholders and experts. To facilitate interaction (and get valuable feedback) various tools can be used depending on the project—such as conferences, workshops, an informative web site—all inviting discussion and input, not passive ‘show and tell’.
6. If translations involve disciplinary-specific language and new concepts, take care with online translation tools.	The advent of new translation technology has been a great boon to transnational and multilingual research, but the emergence of new concepts in science take time to reach an AI tool (at least with good embedded knowledge). The subtleties of conversation across boundaries can be corrupted. This can result in major misunderstandings so when employing such tools, it is important for relevant team members to use a variety of means to check and edit before notes are disseminating to the team. A good dictionary within the team can also help!
